# Identification of key genes and signaling pathways based on transcriptomic studies of aerobic and resistance training interventions in sarcopenia in SAMP8 mice

**DOI:** 10.1016/j.smhs.2024.01.005

**Published:** 2024-02-15

**Authors:** Lunyu Li, Xiaotian Guan, Ying Huang, Bo Qu, Binyu Yao, Haili Ding

**Affiliations:** a School of Sports Medicine and Health, Chengdu Sport University, Chengdu, China; b Institute of Sports Medicine and Health, Chengdu Sport University, Chengdu, China

**Keywords:** Resistance training, Aerobic training, Sarcopenia, Transcriptome

## Abstract

We examined the effects of resistance and aerobic exercise on the gene expression and biometabolic processes of aging skeletal muscle in senescence-accelerated mouse/prone 8 mice, a model of sarcopenia, and compared them with senescence-accelerated mouse/resistant 1 mice acting as controls. We found that exercise improved muscle strength, endurance, fiber size, also modulated genes and pathways related to synaptic transmission, potassium transport, JAK-STAT signaling, and PI3K-Akt signaling. Our results suggested that BDNF, JAK2, RhoC, Myh6, Stat5a, Tnnc1, and other genes may mediate the beneficial effects of exercise on sarcopenia through these pathways.

## Abbreviations

C: Control
M: Model Group
E: Aerobic Training Group
R: Resistance Training Group
AMPK: AMP-Activated Protein Kinases
Akt: protein Kinase B
cGMP: cyclic Guanosine Monophosphate
DEGs: Differentially Expressed Genes
FoxO3: Forkhead Box Protein
GA: Gastrocnemius
GO: Gene Ontology
HE: Hematoxylin–Eosin
IGF-1: Insulin-like growth factor 1
mRNA: messenger Ribonucleic Acid
RNA: Ribonucleic Acid
cDNA: complementary Deoxyribonucleic Acid
DNA: Deoxyribonucleic Acid
JAK: Janus Kinase
KEGG: Kyoto Encylopaedia of Genes and Genomes
Myh6: Recombinant Myosin Heavy Chain 6
mTORC1: mammalian Target of Rapamycin C 1
NTF: Neurotrophic Factor family
NOD: Nucleotide-binding Oligomerization Domain
PI3K: Phosphoinositide-3 kinase
STAT: Signal Transducer and Activator of Transcription
Trk B: Tyrosine kinase receptor B
Tnnc1: Recombinant Troponin C Type 1
UCP3: Uncoupling protein 3
VL: Vastus lateralis
cGMP-PKG: cyclic Guanosine Monophosphate-Protein Kinase G
AGE-RAGE: Advanced Glycosylation End products- Receptor of AGEs
SAMP6: Senescence-Accelerated Mouse/prone6
SAMP8: Senescence-Accelerated Mouse/prone8
SAMR1: Senescence-Accelerated Mouse/resistant 1
BDNF: Brain-derived Neurotrophic Factor
Rap1: Ras-proximate-1
RhoC: Ras homolog gene family member C
CNS: Central Nervous System
CNN3: Calponin 3
E3: Three families of ubiquitin-protein ligases
SMN: Survival Motor Neuron
*SD*: Standard Deviation

## 1 Introduction

Age is an important factor contributing to changes in muscle mass and strength. The progressive decline in muscle mass and strength during the aging process steadily increases the risk of developing sarcopenia.^1^ An age-related degenerative disease that increases the risk of falls, disability, and mortality in older adults. Sarcopenia has a significant impact on physical and mental health and quality of life, as well as imposes a substantial economic burden on social health services. Adequate muscle strength is essential for maintaining functional mobility in older adults and reducing public healthcare expenditures.

A growing body of evidence suggests that exercise interventions can effectively treat skeletal muscle functional deficits.^2^ The use of exercise as an environmentally friendly, safe, and effective method for preventing and treating sarcopenia has become a topic of significant interest in both domestic and international research. Research has determined that a reduction in physical activity is a major risk factor for the development of sarcopenia.^3^ A decrease in physical activity or prolonged bed rest due to illness can result in an inevitable decline in muscle strength and quality in older adults. This decline leads to a loss of elasticity in muscle fibers, muscle hardening, and decreased water content as well as increased intercellular fluid in muscles, causing muscle fiber hypofunction. As a result, individuals may experience muscle fatigue, back pain, leg pain, and other symptoms that further limit limb activity and create a vicious cycle of lack of exercise increasing sarcopenia and increased sarcopenia further limiting exercise. Aerobic and resistance training are considered effective means of preventing these sarcopenia-related conditions.^4^

Research has demonstrated that the development of sarcopenia is associated with several factors, including neuromuscular decline, an imbalance between protein synthesis and catabolism, mitochondrial and chromosomal damage, apoptosis, inflammatory factors, and impaired satellite cell repair.^5^^,^^6^ This suggests that possible molecular mechanisms involving AMP-activated protein kinases (AMPK),^7^ forkhead box protein O3 (FoxO3),^8^ and uncoupling protein 3 (UCP3) lead to reduced resistance to inflammation and oxidative stress. Decreased insulin-like growth factor 1 (IGF-1) expression downregulates sarcoplasmic reticulum calcium rhodopsin release, leading to reduced muscle contraction and cellular pathway signaling. However, the current research lacks focus and clarity.

This study aimed to clarify the effectiveness of aerobic and resistance training in sarcopenia prevention and reduction and identify the pathways and key targets of action in sarcopenia intervention. First, we established a sarcopenia model using senescence-accelerated mouse/prone 8 (SAMP8) prematurely aged mice^9^ and exposed them to several exercise training regimens. Second, after observing the improvement of sarcopenia in SAMP8 mice through aerobic and resistance training, we sequenced the skeletal muscle using transcriptome to investigate the targets and pathways related to the improvement.

## 2 Materials

### 2.1 Ethical approval and development of murine model

Male senescence-accelerated mouse/resistant 1 (SAMR1) and SAMP8 mice 7 months of age were purchased from the Department of Laboratory Animal Science, Peking University School of Medicine (license number SCXK [Beijing] 2016-0010). Animal feeding and training were conducted in the Animal Experiment Center of Chengdu Sport University (Ethics Committee of Chengdu Sport University license number 39, 2021). The laboratory room temperature was between 20 ​°C and 25 ​°C and controlled with light-controlled simulation of a day/night cycle, good ventilation, and ambient humidity for 12 ​hours (h)/day. The mice had free access to standard specific-pathogen-free-grade full-price solid nutrient feed and filtered warm water. The litter tray and animal laboratory were cleaned daily.

### 2.2 Experimental grouping

SAMR1 mice and SAMP8 mice were raised in a standard environment to 28 weeks of age. A blank control group for the SAMR1 mice (group C) and a control group for the SAMP8 mice (group C) were established (*n* ​= ​8 mice/group). The SAMP8 mice were further randomly divided into a model group (group M), a resistance training group (group R), and an aerobic training group (group E) according to the table of random digits method (*n* ​= ​8/group). Group R underwent an 8-week-long intervention during which behavioral tests were conducted and recorded weekly.

### 2.3 Determination of sarcopenia

Sarcopenia was determined using the following tests. (1) Grip strength test: Once a week, the grip strength of the four limbs of all the groups of the SAMP8 mice was tested by a mouse grip strength meter (YLS-13A). (2) Rotarod test: Once a week, the duration that all groups of the SAMP8 mice could stay on a mouse rotarod device set to 300 ​seconds (s) from 4 to 40 rotation per minute (rpm) before falling off was recorded to measure muscle endurance. (3) Wet weight calculation: After the intervention of the SAMP8 mice had been completed, samples were taken by surgery, the mass of the triceps brachii of the mouse calf was recorded, the wet weight ratio of the mouse skeletal muscle was calculated with the recorded body weight, and the muscle mass was evaluated.

### 2.4 Exercise protocol

#### 2.4.1 Aerobic exercise program

The adaptive exercise intervention was performed for 3 days with 10 ​minutes (min) of training per day at a speed of 9 ​m/min and 2 days of rest. Formal training was 5 days/week, 1 session/day for a total of 8 weeks. Weeks 1 and 2 running table running speed was 12 ​m/min for 30 ​min, week 3 was 15 ​m/min for 30 ​min, week 4 was 15 ​m/min for 45 ​min, and weeks 5–8 was 15 ​m/min for 60 ​min at a running table slope of 0°. Training was conducted every Monday to Friday with 2 days of rest. Animal health was checked after each experiment and body weight was measured and recorded weekly during the training period.

#### 2.4.2 Resistance training program

The resistance training adopted the weight-bearing ladder-climbing exercise method, and the adaptive exercise intervention was conducted for 3 days. The climbing ladder (1 ​m height; 2 ​cm interval between steps; and 85° angle between ladder and horizontal plane) was independently developed by our experimental group (Patent No. ZL201821692962.8). The exercise was carried out four times as one set, three sets per day, and 3 days per week. Weight-bearing exercise started from 50% of body weight. During weeks 1 and 2, training was conducted at 50% of body weight. Starting from week 3, training increased by 15% every week until reaching 80%. Training was conducted every Monday, Wednesday, and Friday, and each training session lasted for 40–50 ​min. Animal health was checked after each training session and weight was recorded every week.

### 2.5 Sample collection

After the final intervention, the mice in all the groups fasted for 24 ​h with free access to water. Intraperitoneal injection of 1% pentobarbital sodium (0.1 mL/100 ​g) was administered under anesthesia. Surgery was performed in the laboratory of Chengdu Sport University. Vastus lateralis (VL) and gastrocnemius (GA) muscles of the left and right sides were removed and stored at −80 ​°C. For the left triceps surae, the fascia and fat were removed before the tissue was fixed with 4% paraformaldehyde for histomorphological examination. For the VL muscle, the fascia and fat were removed before the tissue was cut into pea-shaped pieces and placed in a cryopreservation tube. After numbering, the VL muscle was temporarily stored in liquid nitrogen in a −80 ​°C refrigerator for subsequent use for transcriptome sequencing.

### 2.6 Histopathology

In the muscle fiber cross-sectional area test, the main steps of measurement of the cross-sectional area of the triceps surae by hematoxylin and eosin (HE) staining were as follows. (1) The triceps surae fixed in 4% paraformaldehyde was embedded in paraffin and sliced into 4-μm-thick slices. (2) For dewaxing, the slices were first heated at 64 ​°C in an oven for 1 ​h and then dewaxed with xylene three times for 30 ​min each time. (3) The slices were transferred to absolute and 95% ethanol for sequences of dehydration with each concentration for 5 ​min (4) The slices were transferred to 80%, 70%, and 50% ethanol in sequences of dehydration for 5 ​min at each concentration before transfer to distilled water for 3 ​min (5) The slides were stained with hematoxylin for 1 ​min and briefly immersed in tap water to wash off excess staining solution. (6) The slices were immersed in 0.5% hydrochloric acid alcohol for 10 ​s for differentiation (prepared with 70% alcohol). (7) The slices were returned to blue in ammonia water (0.05% lithium carbonate) for 10 ​s, washed with running water for 20 ​min while continuing to return to blue, and transferred to 80% ethanol for 5 ​min (8) The slices were stained for 1 ​min with eosin staining solution (95% ethanol), and (9) transferred to 80%, 85%, 90%, and 95% ethanol and dehydrated for 5 ​min at each concentration; next, the slices were dehydrated in absolute ethanol twice for 5 ​min each time. (10) The transparency of the slides was ensured by maintaining them in xylene for 5 ​min before drying them and sealing them with neutral resin.

### 2.7 Transcriptome sequencing

After modeling intervention, 4 biological replicates of transcriptome sequencing were performed on muscle samples from groups M and R. Total RNA from muscle tissue was assessed using the RNA Nano 6000 Assay Kit of the Bioanalyzer 2100 system (Agilent Technologies, Snata Clara, CA, USA) and RNeasy RNA purification kit (DNase). Total RNA was used as input material for the RNA sample preparations. Briefly, messenger ribonucleic acid (mRNA) was purified from total RNA using poly-T oligo-attached magnetic beads. Fragmentation was conducted using divalent cations under an elevated temperature in the First Strand Synthesis Reaction Buffer (5×). First-strand complementary deoxyribonucleic acid (cDNA) was synthesized using random hexamer primer and M-MuLV Reverse Transcriptase aRNase H. Second-strand cDNA synthesis was subsequently performed using DNA Polymerase I and RNase H. Remaining overhangs were converted into blunt ends via exonuclease/polymerase activities. After adenylation of 3'ends of DNA fragments, adaptors with a hairpin loop structure were ligated to prepare for hybridization. To select cDNA fragments of preferentially 370–420 bp in length, the library fragments were purified with the AMPure XP system (Beckman Coulter, Brea, CA, USA). Polymerase chain reaction (PCR) was performed with Phusion High-Fidelity DNA polymerase, Universal PCR primers, and an Index (X) Primer. Finally, PCR products were purified (AMPure XP system) and library quality was assessed on the Agilent Bioanalyzer 2100 system.

After the library check was qualified, the different libraries were pooled according to the requirements of effective concentration and target data volume. The libraries were then sequenced with an Illumina NovaSeq 6000, and 150 bp paired-end reads were generated. The basic principle of sequencing was sequencing by synthesis. Four fluorescently labeled deoxyribonucleoside triphosphates (dNTPs), DNA polymerase, and adapter primers were added to the sequencing flow cell for amplification. When each sequencing cluster extended the complementary chain, each fluorescently labeled dNTP released the corresponding fluorescence. The sequencer captured the fluorescent signal and converted the light signal into a sequencing peak by computer software to obtain the sequence information of the fragment to be detected.

### 2.8 Differential expression of genes and enrichment analysis

For samples with biological replicates, differential expression of the two comparison sets was performed using DESeq2 software (1.20.0). DESeq2 provides statistical procedures for determining differential expression in digital gene expression data using models based on the negative binomial distribution. The resulting *p* value (*p*_*adj*_) was adjusted using Benjamini–Hochberg correction to control for the false-discovery rates. The criteria *p*_*adj*_ ​≤ ​0.05 and |log2 (fold change) ​≥ ​1 were set as the threshold for significantly differential expression. Gene ontology (GO) enrichment analysis of DEGs was achieved by clusterProfiler (3.8.1) software, in which gene length bias was corrected. GO terms with corrected *p* values lower than 0.05 were considered significantly enriched by DEGs. ClusterProfiler (3.8.1) software was used to analyze the statistical enrichment of DEGs in the Kyoto Encyclopedia of Genes and Genomes (KEGG) pathway, KEGG is a database resource for understanding high-level functions and utilities of biological systems from information at the molecular level, particularly large-scale molecular datasets generated by genome sequencing and other high-throughput databases such as cells, organisms, and ecosystems, was used.

### 2.9 Statistical analysis

Values were presented as the mean ​± ​standard deviation (*SD*). Before statistical analysis, all data were assessed for normality using the one-sample Kolmogorov–Smirnov test. Statistical analysis was performed using GraphPad Prism 9.0 (GraphPad Software Inc., San Diego, CA). Depending on the variable characteristics, the Student's *t*-test (two-sided) or Mann–Whitney *U* test was used to compare the measured parameters between 7- and 9-month-old mice. The differences in relative grip strength, accelerating rotarod test, and mean cross-sectional fiber area of GA muscle among groups C, M, E, and *R* were assessed by one-way analysis of variance (ANOVA) or Kruskal–Wallis *H* tests depending on the variable distribution. *p* ​< ​0.05 were considered statistically significant.

## 3 Results

Comparison of skeletal muscle structure and function before and after aerobic and resistance training.

### 3.1 Changes in relative grip strength

**Fig. 1:**
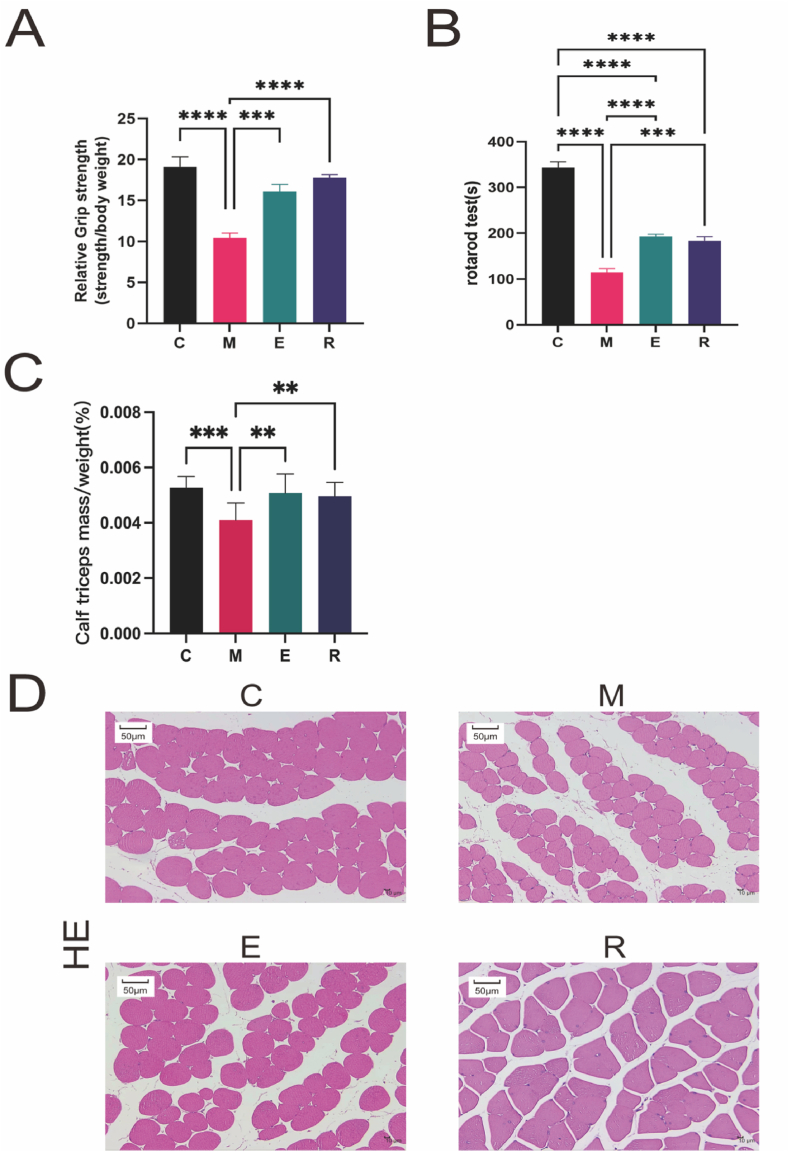
**Comparison of skeletal muscle structure and function in mice before and after training intervention in the control group (C), model group (M), aerobic training group (E), and resistance training group (R).** (A) The results of Relative grip results; (B) The results of the rotating bar test; (C) The results of relative muscle mass; (D) The results of the cross-sectional area of skeletal muscle fibers in each group observed by microscope with Hematoxylin–Eosin (HE) staining.

Relative grip strength results: The body weight of mice in each group remained stable (Table 1). Compared with SAMR1 mice, SAMP8 mice showed a significant decline in relative grip strength before intervention (*p* ​< ​0.01; Table 2). After 8 weeks of aerobic and resistance training, the relative grip strength of mice in group M further declined. Compared with group M, the relative grip strength of group E increased significantly (*p* ​< ​0.05), and the relative grip strength of group R increased more significantly (*p* ​< ​0.01; Table 3). The statistical results of the relative grip strength after 8 weeks of aerobic and resistance training showed that the relative grip strength of mice in group C showed a progressive decline with time while the relative grip strength of groups E and R remained relatively stable after aerobic and resistance training intervention (Fig. 1A). The results indicate that both aerobic and resistance training had a positive effect on the relative grip strength of SAMP8 mice. Compared to group M, which showed a further decline in relative grip strength after 8 weeks, the relative grip strength of mice in the aerobic training group increased significantly, and the relative grip strength of mice in the resistance training group increased even more significantly. Additionally, whereas the relative grip strength of mice in the control group showed a progressive decline over time, the relative grip strength of mice in both the aerobic and resistance training groups remained relatively stable after intervention. These results suggest that both aerobic and resistance training may be effective in improving muscle function in SAMP8 mice.

**Table 1:** Changes in body weight of mice in each group of 8-week training in the control group (C), model group (M), aerobic training group (E), and resistance training group (R).

Time	C	M	E	R
28 weeks	21.61 ​± ​1.54∗∗∗∗	16.73 ​± ​1.55	16.67 ​± ​2.41	16.88 ​± ​1.91
29 weeks	17.62 ​± ​2.32∗	13.65 ​± ​2.60	15.08 ​± ​2.44	13.54 ​± ​0.88
30 weeks	17.99 ​± ​2.59	15.10 ​± ​2.33	16.13 ​± ​2.29	15.31 ​± ​0.99
31 weeks	18.08 ​± ​2.31	11.82 ​± ​1.44	15.78 ​± ​1.89	14.71 ​± ​2.25
32 weeks	18.00 ​± ​3.20	11.00 ​± ​0.86	17.10 ​± ​2.71	16.05 ​± ​2.26
33 weeks	18.62 ​± ​3.50	10.85 ​± ​1.03	17.03 ​± ​1.02	17.98 ​± ​0.88
34 weeks	19.10 ​± ​2.56	10.30 ​± ​0.92	15.56 ​± ​2.45	17.40 ​± ​1.57
35 weeks	19.12 ​± ​3.01	10.40 ​± ​1.48	16.10 ​± ​2.12	17.75 ​± ​0.96

Note: Data shown as means ​± ​standard deviation unless specified otherwise. C-Control, M-Model Group, E-Aerobic Training Group, R-Resistance Training Group. Compared with group M.∗*p* ​< ​0.05, ∗∗∗∗: *p* ​< ​0.000 1.

**Table 2:** Changes in accelerated bar rotation results for each group for 8 weeks training in the control group (C), model group (M), aerobic training group (E), and resistance training group (R).

Time	C	M	E	R
28 weeks	387.0 ​± ​14.276∗∗∗∗	223.89 ​± ​65.74	233.06 ​± ​61.91	220.49 ​± ​71.1
29 weeks	321.61 ​± ​25.43∗∗∗∗	160.17 ​± ​17.37	211.91 ​± ​38.52	195.27 ​± ​48.11
31 weeks	340.22 ​± ​35.79∗∗∗∗	121.61 ​± ​50.43	198.83 ​± ​44.41∗	186.78 ​± ​55.41
33 weeks	342.11 ​± ​33.04∗∗∗	148.46 ​± ​26.56	205.94 ​± ​46.24∗	196.89 ​± ​54.56
35 weeks	343.56 ​± ​29.93∗∗∗∗	114.22 ​± ​21.35	192.89 ​± ​11.49∗∗	183.84 ​± ​21.50∗∗∗∗

Note: Data shown as means ​± ​standard deviation unless specified otherwise. C-Control, M-Model Group, E-Aerobic Training Group, R-Resistance Training Group. Comparison with group M.∗: *p* ​< ​0.05, ∗∗: *p* ​< ​0.01, ∗∗∗: *p* ​< ​0.001, ∗∗∗∗: *p* ​< ​0.000 1.

**Table 3:** Changes in relative muscle mass (%) of triceps muscle and the cross-sectional area (um2) of triceps muscle fibers in the control group (C), model group (M), aerobic training group (E), and resistance training group (R).

Study sample	C	M	E	R
**relative muscle mass**	0.005 3 ​± ​0.000 4∗∗∗	0.004 1 ​± ​0.000 6	0.005 1 ​± ​0.000 7∗∗	0.005 0 ​± ​0.000 5∗∗
**Cross-sectional area**	178.40 ​± ​9.50∗∗∗	164.77 ​± ​6.18	178.87 ​± ​3.02∗∗∗	173.84 ​± ​7.06∗

Note: Data shown as means ​± ​standard deviation unless specified otherwise. C-Control, M-Model Group, E-Aerobic Training Group, R-Resistance Training Group. Comparison with group M. ∗: *p* ​< ​0.05, ∗∗: *p* ​< ​0.01, ∗∗∗: *p* ​< ​0.001.

### 3.2 Changes in the endurance of skeletal muscle according to rotarod test results

Compared with SAMR1 mice, SAMP8 mice showed a significant decrease in rotarod duration before intervention (*p* ​< ​0.000 1; Table 3). After 8 weeks of aerobic and resistance training, the rotarod duration of mice in group M further decreased (*p* ​< ​0.000 1). Compared with group M, the rotarod duration of groups E and R significantly increased (*p* ​< ​0.01). The statistical results of the rotarod test of 8 weeks of aerobic and resistance training showed that the rotarod duration of mice in group C progressively decreased with time, whereas the rotarod duration of groups E and R increased from its initial deterioration after aerobic and resistance training intervention (Fig. 1B).

The results of this test indicate that both aerobic and resistance training had positive effects on the rotarod performance of SAMP8 mice. Compared with group C, which exhibited a further decline in rotarod duration after 8 weeks of intervention, groups M, E, and R all showed a significant decrease in rotarod duration, indicating that the overall endurance of SAMP8 mice deteriorated but that the deterioration of groups E and R recovered after intervention. These findings suggest that both aerobic and resistance training may be effective in enhancing the motor function of SAMP8 mice.

### 3.3 Changes in relative muscle mass

According to the muscle relative mass (muscle wet weight/body weight) results, muscle relative mass increased significantly in groups E and R compared with group M (*p* ​< ​0.01) and did not significantly differ from that of group C after aerobic and resistance training (Fig. 1C). This study's findings demonstrate that both aerobic and resistance training positively influence the muscle relative mass of SAMP8 mice. After 8 weeks of intervention, a significant increase in muscle relative mass was observed in mice from both groups E and R compared with group M. Moreover, no significant difference was found between the muscle relative mass of groups E and R compared with group M. These results suggest that both forms of exercise may effectively improve muscle mass in SAMP8 mice.

### 3.4 Changes in cross-sectional area of muscle fibers

Compared with that of group M, the frequency distribution of muscle fibers in groups E and R showed that aerobic and resistance training induced a shift toward larger muscle fibers (Fig. 1D). The cross-sectional area of gastrocnemius muscle fibers in group E was significantly larger than that of group M (*p* ​< ​0.001) and that of group R was significantly larger than that of group M (*p* ​< ​0.05; Supplementary Fig. 1D).

Before intervention, sarcopenia was judged by observable muscle relative strength and muscle endurance decline in 7-month-old male SAMP8 mice used as a sarcopenia model. After 8 weeks of aerobic and resistance training intervention, muscle relative strength, skeletal muscle relative mass, and muscle endurance of the mice had significantly improved.

### 3.5 Comparison of transcriptional profiles in SAMP8 mice with sarcopenia after aerobic and resistance training

#### 3.5.1 Differentially expressed gene screening results

**Fig. 2:**
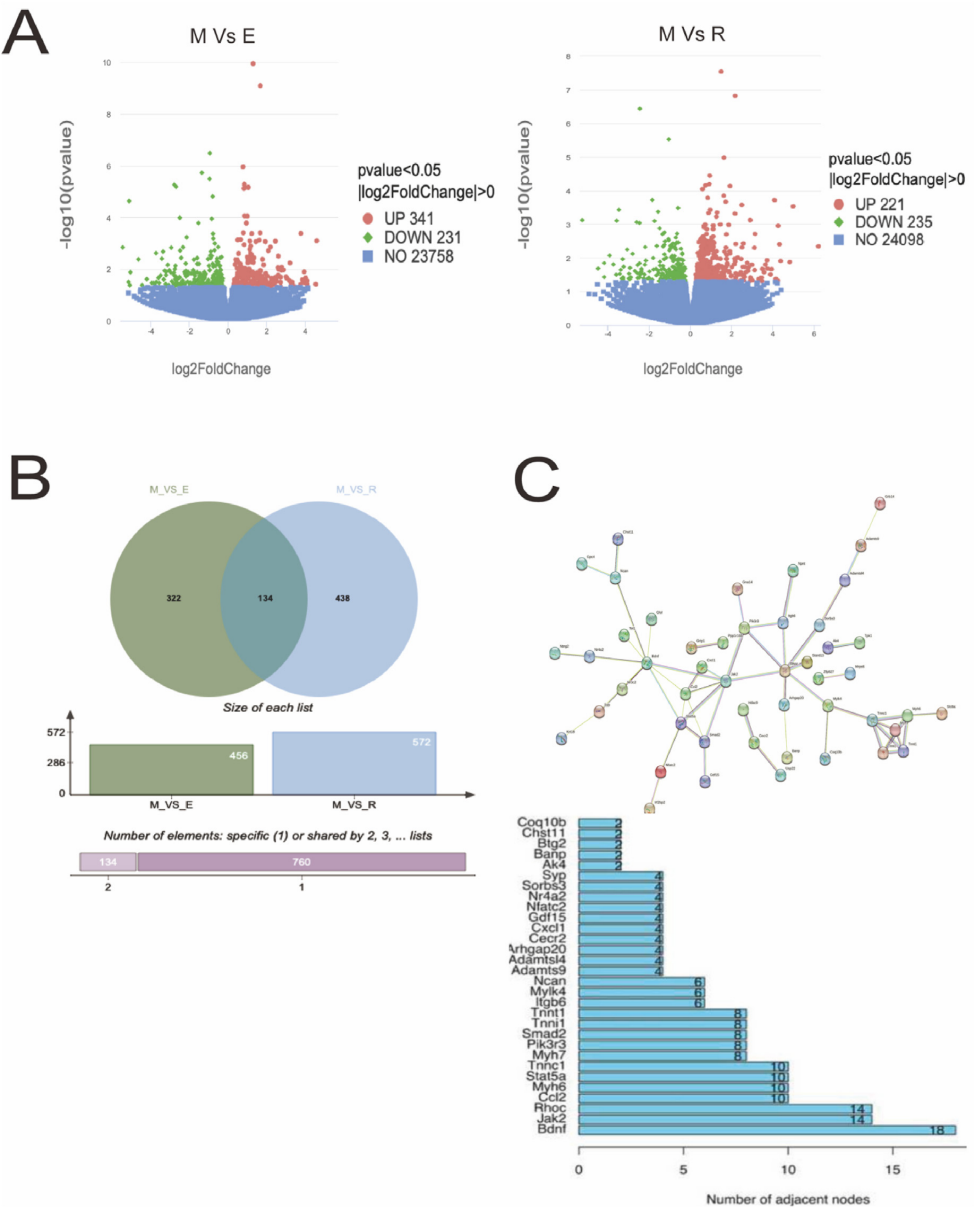
**Comparison of transcriptional profiles in SAMP8 mice with sarcopenia after aerobic and resistance training in the model group (M), aerobic training group (E), and resistance training group (R).** (A) Differentially Expressed Gene Screening; (B, C) Aerobic and resistance training transcriptome core gene screen.

The significant (*p* ​< ​0.05) differences in mRNAs among the groups were analyzed and visualized using a volcano plot. Genes with *p* ​> ​0.05 were labeled in blue, genes with log2FC ​< ​0 with downregulated expression were labeled in green, and genes with log2FC ​> ​0 with upregulated expression were labeled in red. The skeletal muscle gene expression profile data obtained after transcriptome analysis of the aerobic and resistance training and model groups were normalized by the median. The limma package in R language was used for gene screening of the normalized data (Fig. 2A) A total of 456 differential DEGs were screened in the aerobic training and model groups and 572 DEGs in the resistance training and model groups. Compared with the model group, the aerobic training group had a total of 235 mRNAs with upregulated expression and 221 mRNAs with downregulated expression, and the resistance training group had 231 mRNAs with upregulated expression and 341 mRNAs with downregulated expression (log2FC ​≥ ​0, *p* ​< ​0.05).

#### 3.5.2 Comparison of aerobic and resistance training transcriptome core gene data

Wayne plot analysis revealed a total of 456 DEGs in the R vs. M group, 572 DEGs in the E vs. M group, and 134 identical DEGs in the two data sets (Fig. 2B). Differential gene protein interaction network analysis revealed that the DEGs obtained from the previous experiment had been imported into the Search Tool for the Retrieval of Interacting Genes/Proteins (STRING) functional protein association network. The protein–protein interaction (PPI) network obtained from STRING consisted of 110 node proteins and 58 interaction relationships (enrichment *p* ​< ​0.000 1). All filtered protein information was exported and the PPI network data were imported into an R script. Evaluation of proteins based on the number and strength of their interaction relationships resulted in the identification of the core genes BDNF, Jak2, RhoC, Ccl2, Myh6, Stat5a, and Tnnc1 (Fig. 2C).

### *3.6* *GO and KEGG analyses* of differentially expressed genes

**Fig. 3:**
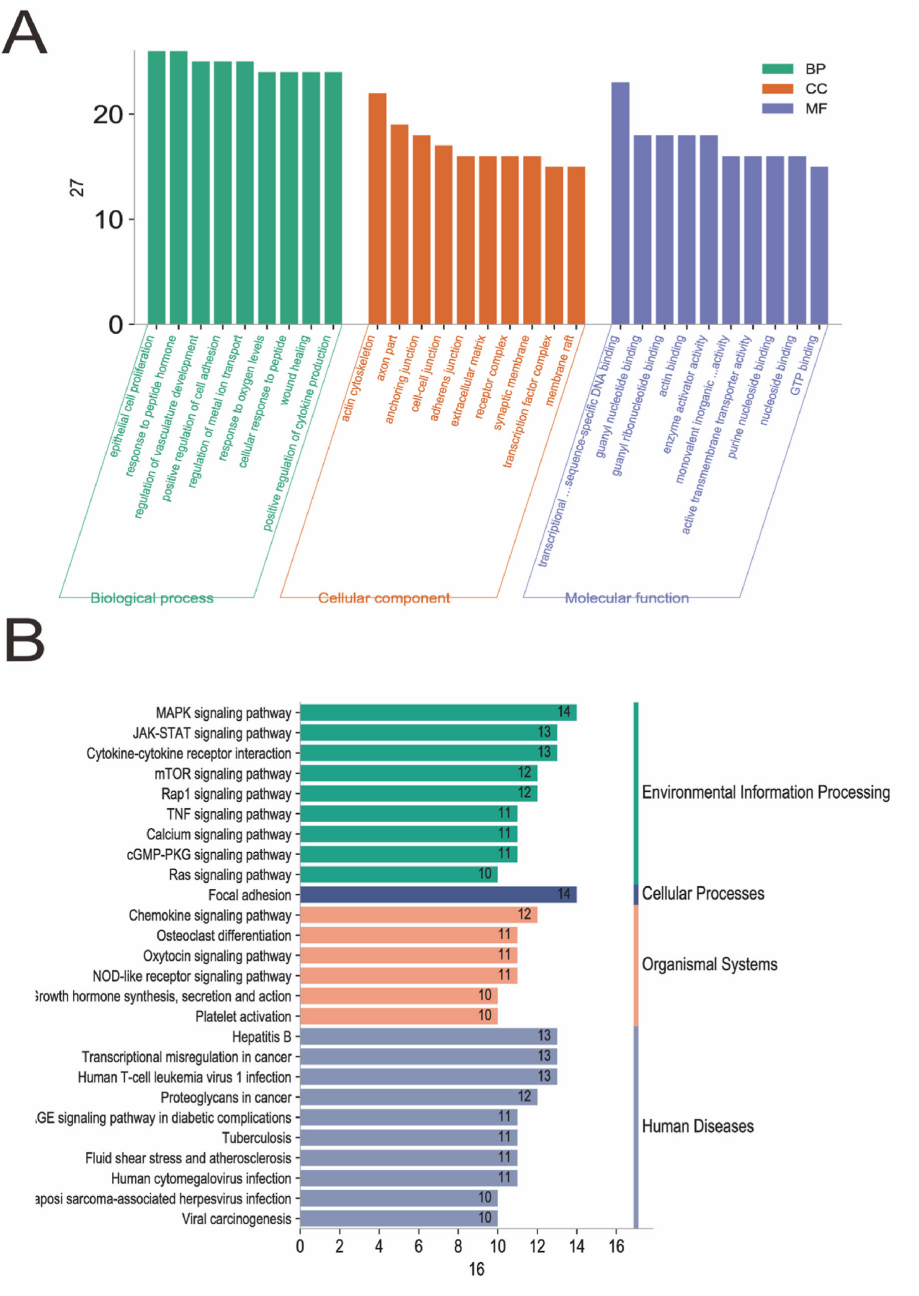
**Transcriptome analysis.** GO enrichment (A) and KEGG pathway (B) were analyzed in the model group (M), and aerobic training group (E).

GO and KEGG analysis of *group* E: The results of GO and KEGG signaling pathway enrichment analysis of total DEGs in group E using the Database for Annotation, Visualization, and Integrated Discovery (DAVID) are shown in Fig. 3A. Compared with group M, the DEGs in group E were primarily involved in skeletal system development, response to nutrient levels, muscle organ development, B cell activation, organelle fission, and blood circulation biological processes. They were primarily localized in the plasma membrane protein complex, plasma membrane outer, transcription factor complex, receptor complex, microtubules, interneuronal synapses, and their molecular functions were involved in receptor regulatory activity, transcriptional activation activity, and sequence-specific DNA binding in transcriptional regulatory regions of RNA polymerase II, adenosine triphosphate-hydrolyzing enzyme (ATPase) activity, inorganic cation transmembrane transporter protein activity, nucleoside binding, and guanylate binding. Further, KEGG analysis showed that DEGs were mainly associated with the phosphoinositide-3-kinase–protein kinase B/Akt (PI3K-Akt) signaling pathway, cell adhesion molecules, cellular senescence, T helper 17 ​cell (Th17) cell differentiation, advanced glycosylation end product-receptor of AGE (AGE-RAGE) signaling pathway in diabetic complications, Janus kinase/signal transducers and activators of transcription (JAK-STAT) signaling pathway, cyclic guanosine monophosphate-protein kinase G (cGMP-PKG) signaling pathway, and ras-proximate-1 (Rap1) signaling pathway (Fig. 3B).

**Fig. 4:**
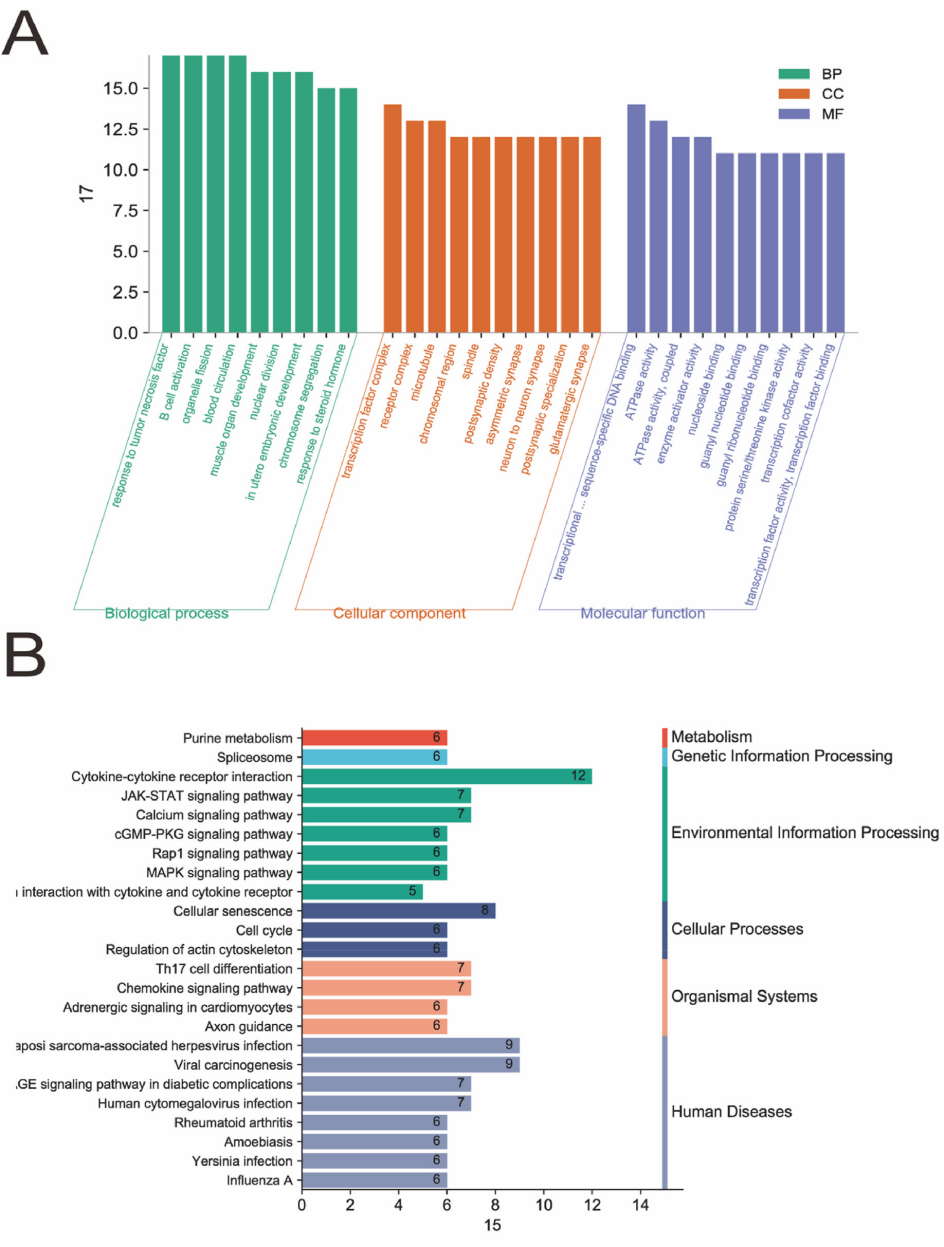
**Transcriptome analysis.** GO enrichment (A) and KEGG pathway (B) were analyzed in the model group (M), and resistance training group (R)**.**

GO and KEGG analysis of group R: The results of GO and KEGG signaling pathway enrichment analysis of total DEGs using the DAVID database are shown in Fig. 4A. Compared with group M, GO enrichment analysis revealed that the DEGs of group R were mainly involved in skeletal system development, response to peptides, embryonic organ development, monovalent inorganic cation transport, and positive regulation of MAPK cascade. They are mainly localized in the actin cytoskeleton, outer plasma membrane, extracellular matrix, adhesion junctions, receptor complex, and synaptic membrane. Their molecular functions are mainly involved in transcriptional activation activity, sequence-specific DNA binding in the transcriptional regulatory region of RNA polymerase II, receptor-ligand activity, guanylate binding, actin binding, enzyme activation activity, monovalent inorganic cation-transporter protein activity, and active transmembrane transporter protein activity. KEGG analysis showed that the DEGs were mainly associated with the PI3K-Akt signaling pathway, MAPK signaling pathway, JAK-STAT signaling pathway, mammalian target of rapamycin (mTOR) signaling pathway, chemokine signaling pathway, Rap1 signaling pathway, AGE-RAGE signaling pathway in diabetic complications, fluid shear stress and atherosclerosis, nucleotide-binding oligomerization domain (NOD)-like receptor signaling pathway, and cGMP-PKG signaling pathway (Fig. 4B).

**Fig. 5:**
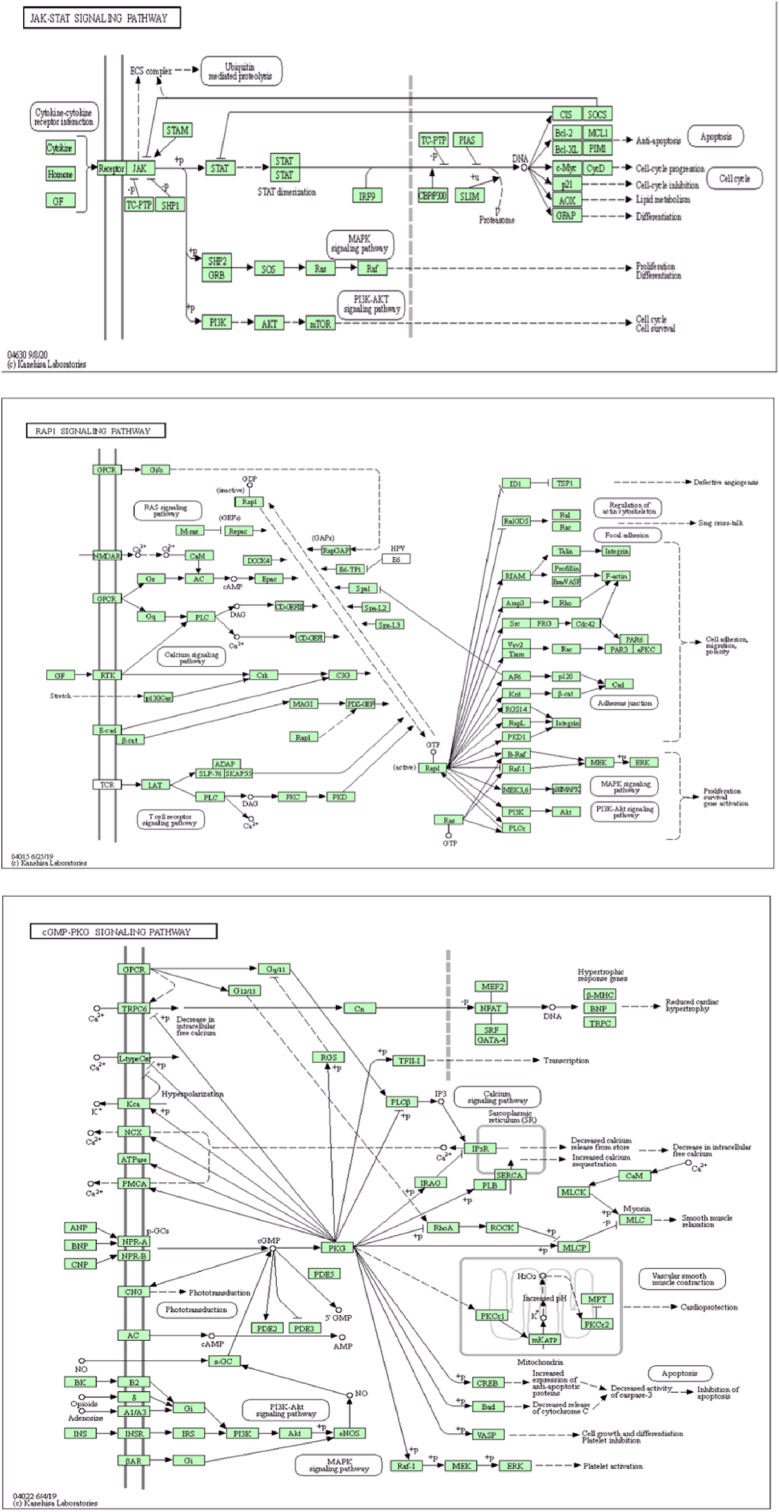
Analysis of common signaling pathways by differential gene KEGG enrichment between different training groups and the model group (M), Janus Kinase-Signal Transducer and Activator of Transcription (JAK-STAT), Ras-proximate-1(Rap1), cyclic Guanosine Monophosphate-Protein Kinase G (cGMP-PKG) signal pathway.

GO enrichment analysis of DEGs revealed that compared with those of group M, the DEGs of groups E and R groups were jointly involved in the development of the skeletal system. KEGG analysis showed that the DEGs in groups E and R groups were jointly enriched in the JAK-STAT, cGMP-PKG, and Rap1 signaling pathways (Fig. 5).

## 4 Discussion

Progressive skeletal muscle atrophy is an age-related aging syndrome characterized by a progressive decline in muscle mass and function. A primary health problem of the aging population, it significantly increases the risk of falls, fractures, and disability; limits activity; and increases the burdens on families and society.^10^ Therefore, it is important to investigate the pathogenesis of age-related skeletal muscle atrophy and its therapeutic targets.

Exercise training is one of the most important, safe, and feasible interventions to delay or even halt the onset of sarcopenia. Exercise can improve physical performance; reduce the risk of falls; and improve balance, cardiorespiratory capacity, and muscle strength.^11^^,^^12^ Recommendations for the appropriate design of exercise programs for frail individuals are not always clear. Despite research showing that long-term adherence to scientifically investigated exercise can enhance skeletal muscle performance and prevent age-related muscle atrophy, few studies have examined the relationship between different exercise training modalities. Aerobic and resistance training are commonly used endurance modalities that have been shown to be effective in improving skeletal muscle mass and function. The recommended physical interventions mainly focus on muscle strength, endurance, balance, and flexibility training. Among them, aerobic exercise and resistance training, especially resistance training, are considered important components of strength training for frail populations.^13^ In fact, resistance training, alone or in combination with other training components, can improve muscle mass, muscle strength and function, and functional capacity.^14^^,^^15^

SAMP8 is the most commonly used accelerated aging mouse model in sarcopenia research. Compared with SAMP6 and SAMR1, SAMP8 mice show typical features of muscle aging at a relatively young age at a rate almost twice that of other models.^16^ Many studies have used SAMP8 mice to verify the effect of exercise intervention on sarcopenia and evaluate its effects.^17^^,^^18^ Using the SAMP8 model to test the effect of long-term exercise on preventing sarcopenia, Takigawa et al. found that long-term spontaneous physical exercise may help recovery from age-related sarcopenia.^17^ In an investigation of the muscle mass and structural and functional characteristics of the GA muscle, Guo et al. found that SAMP8 mice had the largest muscle mass in the GA muscle at 7 months, followed by a downward trend.^19^

Although the pathogenesis of age-related skeletal muscle atrophy remains unclear, several possible mechanisms have been proposed. Research has indicated that the pathogenesis of age-related skeletal muscle atrophy may be associated with several factors. Among these, decreased physical activity is considered the most significant risk factor for the development of age-related skeletal muscle atrophy.^20^ The number of muscle fibers tends to decrease with age, but this decrease is significantly less pronounced with maintenance of a certain level of physical activity compared with remaining sedentary. Hormonal changes associated with aging also play a role in age-related skeletal muscle atrophy. A decrease in the secretion of hormones related to metabolic synthesis can result in the loss of skeletal muscle fibers. As the body's ability to synthesize protein declines with age, a reduction in protein intake can lead to a decrease in muscle mass. The accumulation of oxidized proteins in skeletal muscle results in the deposition of lipofuscin and cross-linked proteins that cannot be adequately removed by the hydrolysis system, leading to a decrease in skeletal muscle strength.^21^ In addition, altered motor unit structure, genetics, and early development play a role in aging skeletal muscle atrophy.

Exercise intervention can modulate changes in gene expression associated with aging to alter skeletal muscle phenotypes and prevent sarcopenia. Through DEG and PPI network analysis, this study aimed to explore the pathogenesis of aging skeletal muscle atrophy and the role of different exercise intervention modalities. Utilizing bioinformatics to screen the skeletal muscle transcriptome of SAMP8 mice in aerobic, resistance training, and anti-aging groups, 6 key genes involved in aerobic and resistance training—BDNF, JAK2, RhoC, Myh6, Stat5a, and Tnnc1— were identified. These genes may be candidate targets for the clinical prevention and treatment of sarcopenia. The roles of proteins encoded by these key genes in this regulatory network and their potential as targets for clinical improvement of senescent skeletal muscle atrophy require further investigation through extensive basic experiments.

Among the genes, BDNF showed the highest relevance in differential genetic screening. A member of the neurotrophic factor family (NTF), BDNF is expressed in the central nervous system (CNS) and plays a regulatory role in cellular function by activating tyrosine kinase receptor B (Trk B). Exercise increases BDNF expression in the CNS, blood, and skeletal muscle.^22^ A skeletal muscle contraction-induced derivative protein, BDNF can increase fat oxidation in skeletal muscle through the AMPK pathway and may be a possible therapy for metabolic diseases.^23^

JAK2 is a member of the Janus kinase/signal transduction and transcriptional activation molecule signaling pathway, which is widely involved in physiological processes such as proliferation, differentiation, apoptosis, and inflammation. In this study, the JAK/STAT pathway showed the highest KEGG enrichment, and JAK2 expression had a dual effect: its inhibition reduced inflammatory oxidation and thus decreased the ablation of skeletal muscle while its activation also reduced muscle ablation.^24^^,^^25^ Activation of JAK2 has been found to inhibit apoptosis of muscle stem cells, thereby controlling muscle growth.^26^ A non-receptor tyrosine kinase that consists of the FERM region, N-terminal receptor-binding region, V617F pseudokinase region, and JH1 kinase region, JAK2 regulates change in the expression of genes involved in skeletal muscle development.

RhoC belongs to the Rac subfamily of small signaling proteins and plays an important role in the organization of the actin skeleton, cell morphogenesis, and cell motility.^27^ RhoC can affect the degradation of the extracellular matrix at the end of skeletal muscle differentiation, leading to impaired cell fusion.^28^ In addition, RhoC interacts with Rock1 to negatively regulate muscle differentiation and myotube fusion by activating calponin 3 (CNN3) through phosphorylation.^28^ These findings demonstrate that changes in RhoC expression can impact myotube formation.^29^ Myh6 is present only in slow muscle fibers, and its expression regulates contraction in skeletal muscle.^30^ Stat5a prolactin increases survival motor neuron (SMN) expression and survival via the STAT5 pathway.^31^^,^^32^ In a mouse model of severe spinal muscular atrophy, murine skeletal muscle growth and fiber composition were regulated via the transcription factor STAT5a/b, linking growth hormone to the androgen receptor. Tnnc1 is expressed in slow-acting and cardiac muscle tissue and is required for skeletal muscle fiber integrity and energy metabolism.^33^

As 3 families of ubiquitin-protein ligase (E3) ubiquitin ligase expression are downregulated in aging skeletal muscle, aging can lead to significant changes in skeletal muscle gene expression profiles. However, exercise intervention can modulate these changes to alter skeletal muscle phenotypes. In this study, analysis of a sarcopenia model established in SAMP8 mice revealed that aerobic and resistance training interventions can improve skeletal muscle mass and function. Transcriptomic analysis identified BDNF, JAK2, RhoC, Myh6, Stat5a, and Tnnc1 as core target genes that may mediate the effects of different exercise intervention stimuli through the JAK-STAT, cGMP-PKG, and Rap1 pathways to improve the skeletal muscle phenotype of sarcopenia. The mechanisms by which these pathways act in aging skeletal muscle in response to exercise intervention have yet to be fully elucidated. However, the mammalian target of rapamycin complex 1 (mTORC1), a key signaling molecule in skeletal muscle that responds to anabolic stimuli and regulates protein synthesis and adaptive hypertrophy, may be a common pathway of action. Further research is needed to fully examine these genes and pathways and elucidate the mechanisms by which exercise intervention affects gene expression and skeletal muscle function in aging individuals.

## 5 Conclusions

This study investigated the effects of aerobic and resistance training on sarcopenia in SAMP8 mice, a widely used model of accelerated muscle aging, by measuring muscle strength, mass, and function and comparing these measures with those of SAMR1 mice, a resistant strain. Both types of exercise improved outcomes in SAMP8 mice and the BDNF, Jak2, RhoC, Ccl2, Myh6, Stat5a, Tnnc1, and JAK/STAT pathways emerged as common molecular targets and pathways involved in exercise-induced benefits. These findings suggested that exercise training could be a safe and effective intervention to prevent or reverse sarcopenia in aging mice.

## Submission statement

The manuscript has not been published and is not under consideration for publication elsewhere. All authors have read and agree with the manuscript content, and the manuscript will not be submitted elsewhere for review and publication.

## Ethical approval statement for animal use

Male SAMR1 and SAMP8 mice (7 months of age) were purchased from Peking University School of Medicine (Department of Laboratory Animal Science), license number: SCXK (Beijing) 2016-0010. Animal feeding and training were carried out in the Animal Experiment Center of Chengdu Sport University (license number: Ethics Committee of Chengdu Sport University 2021 No. 39). The laboratory room temperature was controlled at 20 °C-25 ​°C, and a good ventilation environment and ambient humidity were guaranteed for 12 ​h. Lights on and lights off simulated day and night alternation. The mice had free access to standard SPF-grade full-price solid nutrient feed and filtered warm water. The litter tray and animal laboratory were cleaned daily.

## Authors’ contributions

DH: conceptualization, methodology, validation, writing—original draft preparation, and project administration. LL and GX: software, formal analysis, investigation, resources, supervision, and funding acquisition. LL, HY, YB, and QB: writing—review and editing. All authors have read and agreed to the published version of the manuscript.

## Funding

Funding was received from the National Natural Science Foundation of China (NSFC) (NSFC Grant No. 81904318), Sichuan Science and Technology Program (22ZYZYTS0046), Key Laboratory of Sports Medicine of Sichuan Province, Institute of Sports Medicine and Health, Chengdu Sport University (No. 2021-A030), and the National Key Research and Development Program of China (No. 2018YFF0300904), Sichuan Province central government guides local science and technology development project (2022ZYD0062), Sichuan Province science and technology innovation and entrepreneurship seedling project (MZGC20230033).

## CRediT authorship contribution statement

**Lunyu Li:** Writing – original draft, Supervision, Software, Resources, Methodology, Funding acquisition, Formal analysis, Data curation. **Xiaotian Guan:** Writing – original draft, Supervision, Software, Resources, Investigation, Funding acquisition, Formal analysis. **Ying Huang:** Visualization, Validation, Software, Formal analysis. **Bo Qu:** Visualization, Software, Methodology. **Binyu Yao:** Supervision, Software, Formal analysis. **Haili Ding:** Writing – review & editing, Project administration, Conceptualization.

## Conflict of interest

There no conflicts of interest.
