# Supplementary material for: Identification of key genes and signaling pathways based on transcriptomic studies of aerobic and resistance training interventions in sarcopenia in SAMP8 mice
Source: Sports Med Health Sci. 2024 Feb 15;6(4):358–69. doi: 10.1016/j.smhs.2024.01.005 (PMC11411317; doi:10.1016/j.smhs.2024.01.005)
Supplement: Multimedia component 1 [file mmc1.docx]

Supplementary fig 1. **Comparison of skeletal muscle structure and function in mice before and after training i**n the control group (C), model group (M), aerobic training group (E), and resistance training group (R).

**(**A) Baseline results for relative grip strength; (B) Baseline results of the rotating bar test; (C) Baseline results of relative muscle mass; (D) The cross-sectional area results of skeletal muscle fibers.**
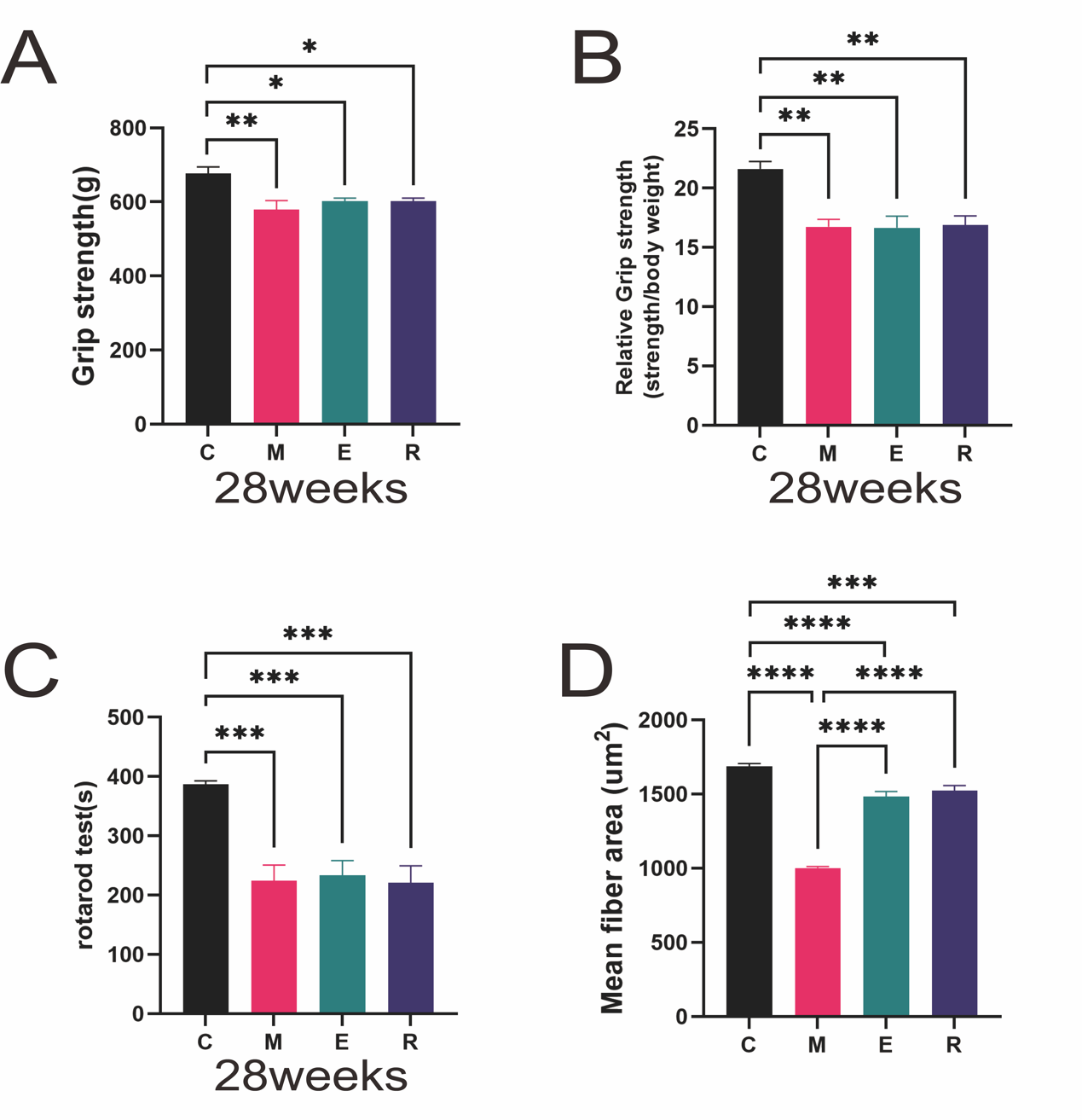
**
